# Supplementary material for: A Global Screen for Assembly State Changes of the Mitotic Proteome by SEC-SWATH-MS
Source: Cell Syst. 2020 Feb 26;10(2):133–155.e6. doi: 10.1016/j.cels.2020.01.001 (PMC7042714; doi:10.1016/j.cels.2020.01.001)

P15170 | ERF3A_HUMAN | GSPT1 ERF3A
Monomer MW [kDa]: 55.756 Monomer expected elution fraction: 45

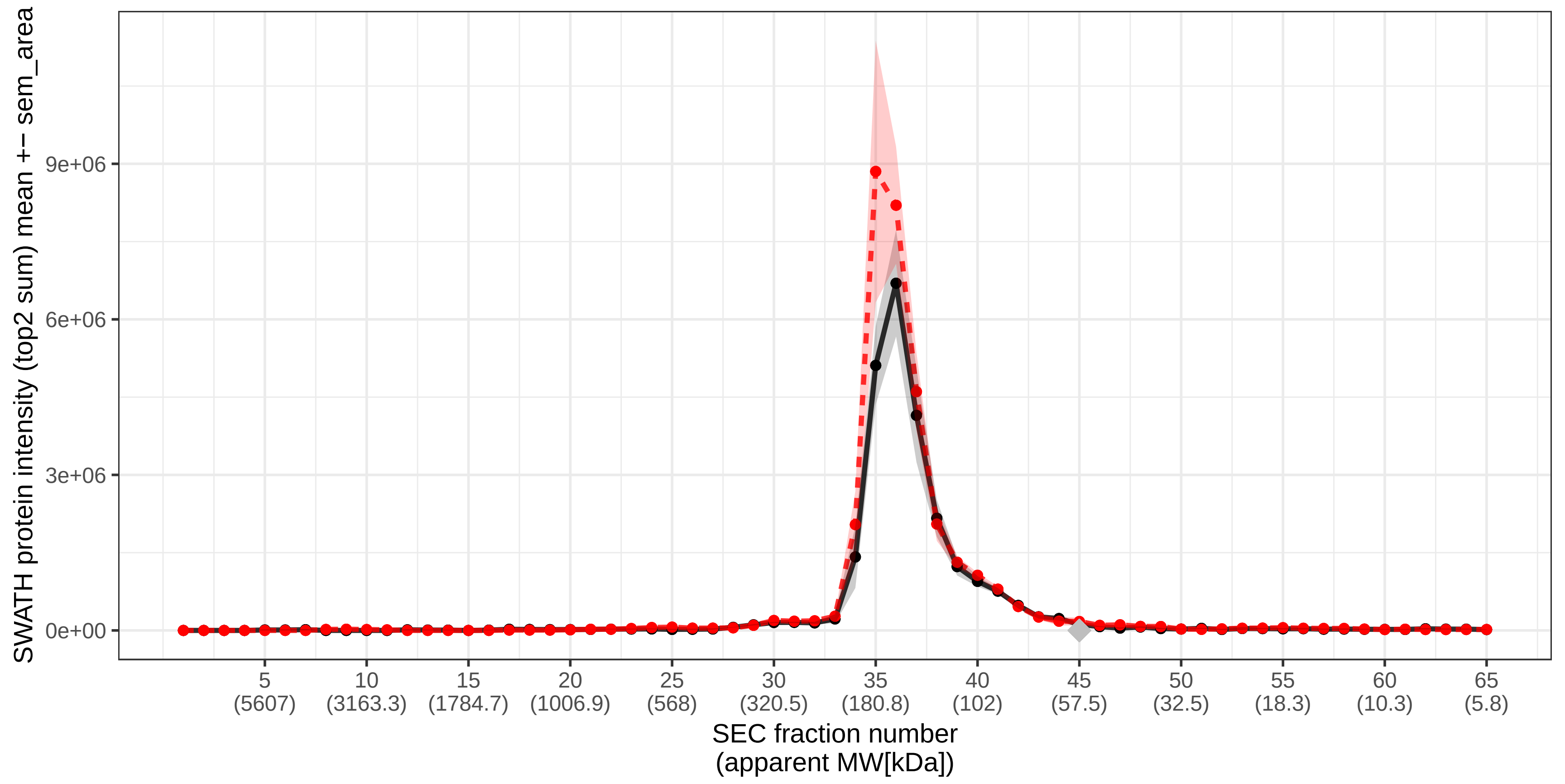

Supplement: Data S1. SEC-SWATH-MS Protein Chromatograms, Related to Figure 1 [file mmc6.zip › SECchrom_P15170_ERF3A_HUMAN_GSPT1_ERF3A.pdf]
